# Monocarboxylate transporter 1 (MCT1), a tool to stratify acute myeloid leukemia (AML) patients and a vehicle to kill cancer cells

**DOI:** 10.18632/oncotarget.20294

**Published:** 2017-08-16

**Authors:** Filipa Lopes-Coelho, Carolina Nunes, Sofia Gouveia-Fernandes, Rita Rosas, Fernanda Silva, Paula Gameiro, Tânia Carvalho, Maria Gomes da Silva, José Cabeçadas, Sérgio Dias, Luís G. Gonçalves, Jacinta Serpa

**Affiliations:** ^1^ Centro de Estudos de Doenças Crónicas da Faculdade de Ciências Médicas da Universidade NOVA (CEDOC-FCM-UNL), Lisbon, Portugal; ^2^ Unidade de Investigação em Patobiologia Molecular do Instituto Português de Oncologia de Lisboa Francisco Gentil (IPOLFG), Lisbon, Portugal; ^3^ Instituto de Tecnologia Química e Biológica António Xavier (ITQB Nova), Oeiras, Portugal; ^4^ Serviço de Hemato-Oncologia, IPOLFG, Lisbon, Portugal; ^5^ Instituto de Medicina Molecular da Universidade de Lisboa, Lisbon, Portugal; ^6^ Serviço de Anatomia Patológica, IPOLFG, Lisbon, Portugal

**Keywords:** metabolic switch, MCT1, lactate, VEGF, BM microenvironment

## Abstract

Dysregulation of glucose/lactate dynamics plays a role in cancer progression, and MCTs are key elements in metabolic remodeling. VEGF is a relevant growth factor in the maintenance of bone marrow microenvironment and it is also important in hematological diseases.

Our aim was to investigate the role of VEGF in the metabolic adaptation of Acute myeloid leukemia (AML) cells by evaluating the metabolic profiles and cell features according to the AML lineage and testing lactate as a metabolic coin.

Our *in vitro* results showed that AML promyelocytic (HL60) and monocytic (THP1) (but not erythroid- HEL) lineages are well adapted to VEGF and lactate rich environment. Their metabolic adaptation relies on high rates of glycolysis to generate intermediates for PPP to support cell proliferation, and on the consumption of glycolysis-generated lactate to supply biomass and energy production. VEGF orchestrates this metabolic network by regulating MCT1 expression. Bromopyruvic acid (BPA) was proven to be an effective cytotoxic in AML, possibly transported by MCT1.

Our study reinforces that targeting metabolism can be a good strategy to fight cancer. MCT1 expression at the time of diagnosis can assist on the identification of AML patients that will benefit from BPA therapy. Additionally, MCT1 can be used in targeted delivery of conventional cytotoxic drugs.

## INTRODUCTION

Cancer metabolism is considered an emerging hallmark in cancer [1, 2] and besides its role in tumorigenesis is still far from being completely known, although its relevance in metabolic adaptation in cancer progression is inescapable. The Warburg effect was the first well documented metabolic adaptive process exhibited by cancer cells. Warburg postulated that the high rate of glycolysis aimed to sustain energetic demands of cancer and despite being an anaerobic metabolic pathway, glycolysis would work on independent of oxygen availability [3–5]. However, which would be the cost for a cell to maintain glycolysis as the main energy source? Would it be profitable and sustainable? There are some experimental evidences that highlight the use of glycolysis by highly proliferative cancer cells [6, 7]. Glycolysis is used mainly to sustain nucleotide synthesis dependent on Phosphate Pentose Pathway (PPP), and to maintain oxidative phosphorylation (OXPHOS) active, supplied by other substrates namely lactate resulting from glycolysis. The metabolic symbiosis which occurs among cancer cells and between cancer cells and other stromal cells [8] requires a tightly coordinated system of monocarboxylate transporters (MCTs) [9, 10] and lactate dehydrohgenases (LDH) [11]. MCTs and LDHs allow the production and export of lactate after glycolysis and afterwards the import and its convertion into pyruvate to supply tricarboxylic acid (TCA) cycle and OXPHOS for energy and biomass production [12]. Accordingly, the increased levels of lactic acidosis in cancer patients can be a consequence not only of cell lysis but also of cell proliferation, in which lactate is transiently secreted and afterwards taken up.

The French-American-British (FAB) classification of Acute myeloid leukemia (AML) organizes AML in categories according to cancer cells lineage and differentiation state [13]; 80% of AML cases derive from a granulocytic and monocytic progenitor (GMP), 15% derive from a megakaryocytic and erytroblastic progenitor (MEP) and the remnant 5% are from basophilic and eosinophilic lineage.

Here, we use AML as a cancer model given that lactic acidosis, although rare, can be a severe condition at diagnosis and relapse. Often and despite not being a clinical lactic acidosis, lower pH levels accompanied by increased levels of lactate in the peripheral blood compared to normal individuals, have been reported in AML patients [14–16]. We hypothesized that leukemia cells take advantage on the bone marrow (BM) natural microenvironment and molecular stimuli, having the metabolic adaptation as the driving force of certain subsets of AML progression. Thus, testing VEGF as a microenvironment regulator and lactate as a metabolic coin, we investigated the differences in metabolic profiles and cell features according to the AML lineage (M0 to M7).

## RESULTS

### Lactate and glucose metabolic profiles differ between cell lines from different AML types

The lactate and glucose catabolism was defined by NMR spectroscopy which is a reliable technique to evaluate metabolic profiles [17]. The use of carbon 13 (^13^C) labelled compounds allows us to follow the unequivocal insertion of those ^13^C in other organic compounds. Hence, as we used ^13^C-lactate or ^13^C-glucose in separate assays to define metabolic profiles, it is clear which compounds are derived from glucose or lactate.

**Figure 1:**
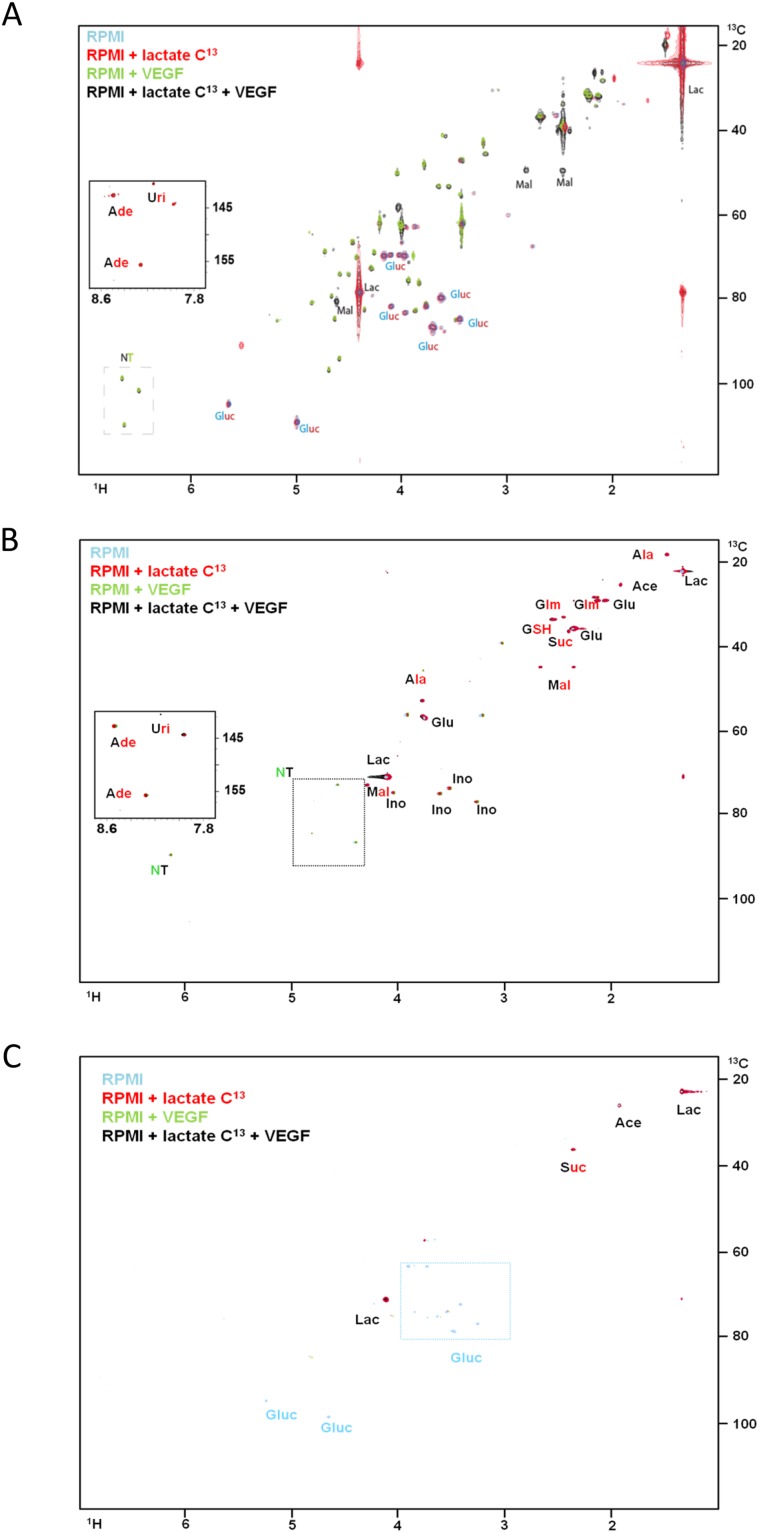

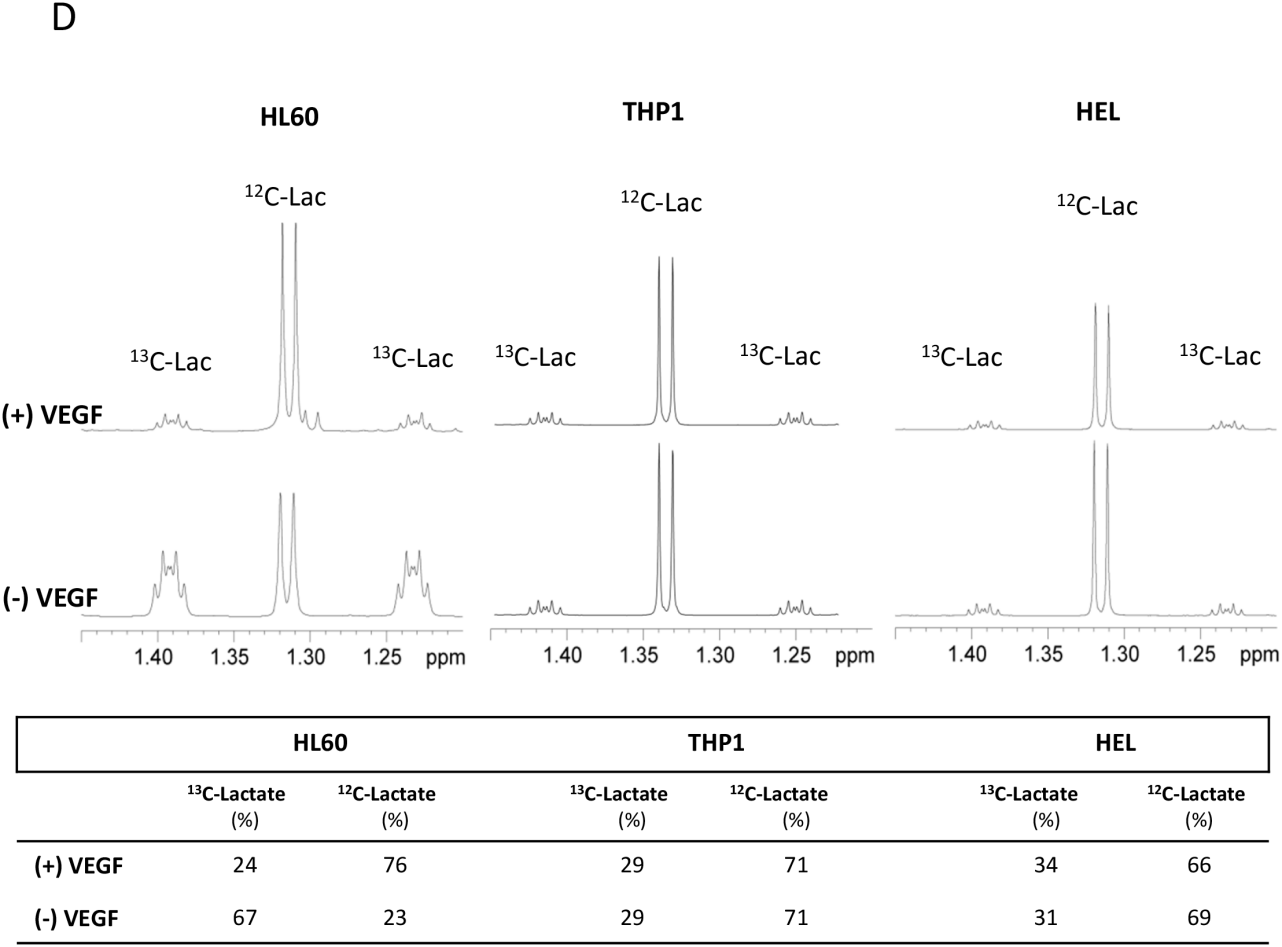
The effect of VEGF in lactate metabolism in AML cell lines ^1^H-^13^C-HSQC NMR spectra of HL60 **(A)**, THP1 **(B)** and HEL **(C)** intracellular extracts cultured in RPMI (blue); RPMI with ^13^C-U-lactate (red), RPMI with 10μg/mL of VEGF (green) and RPMI with ^13^C-U-lactate and 10μg/mL of VEGF (black). **(D)** ^1^H-NMR spectra highlight of the lactate methyl group when the three cell lines (HL60, THP1 and HEL) were cultured with ^13^C-U-lactate: in the absence and in the presence of VEGF. The percentage of intracellular ^13^C-lactate and ^12^C-lactate in each condition is indicated in the board. Gluc- glucose; Ace- acetate; Lac- lactate; Mal- malate; Ala- alanine; Glut- glutamate; Ade- adenosine; Suc- succinate; Ino- inositol; GSH- glutamyl moiety of glutathione and NT- ribosyl moiety of nucleotides. Results were obtained from 3 independent replicates, and representative figures are presented.

In the HL60 (promyelocytic) and THP1 (monocytic) cell lines, lactate was widely used in the synthesis of tricarboxylic (TCA) cycle intermediates (malic acid and acetic acid) and aminoacids (alanine) (Figure 1A and 1B). Both cell lines incorporated lactate ^13^C carbons in the nitrogen purine bases of nucleotides (Figure 1A and 1B). In the presence of VEGF, intracellular glucose was not detected indicating it was consumed, and the resonances of ribosyl moieties of nucleotides are increased in the presence of VEGF without and with ^13^C-lactate (Figure 1A and 1B). Interestingly, in the THP1 cell line it was also possible to see the incorporation of ^13^C from lactate into glutamine and glutamate moiety of GSH, and in succinate (Figure 1B). Regarding erythroblastic cell line, HEL, the metabolism of lactate is restricted to the synthesis of malic acid and proline. VEGF does not affect the metabolic profile of this cell line (Figure 1C).

In addition, it was observed that in the presence of VEGF, the ratio of ^13^C and ^12^C in the lactate was altered in HL60 cell line: in the presence of VEGF the ^12^C-lactate levels increased, from 23% of the total lactate to 76%. The origin of ^12^C-lactate must be the ^12^C glucose available in the culture medium, indicating that VEGF increases the uptake of glucose and the glycolysis rate (Figure 1D). In THP1 and HEL cell lines the *ratio* of ^13^C-lactate and ^12^C-lactate remained the same with and without VEGF stimuli.

**Figure 2:**
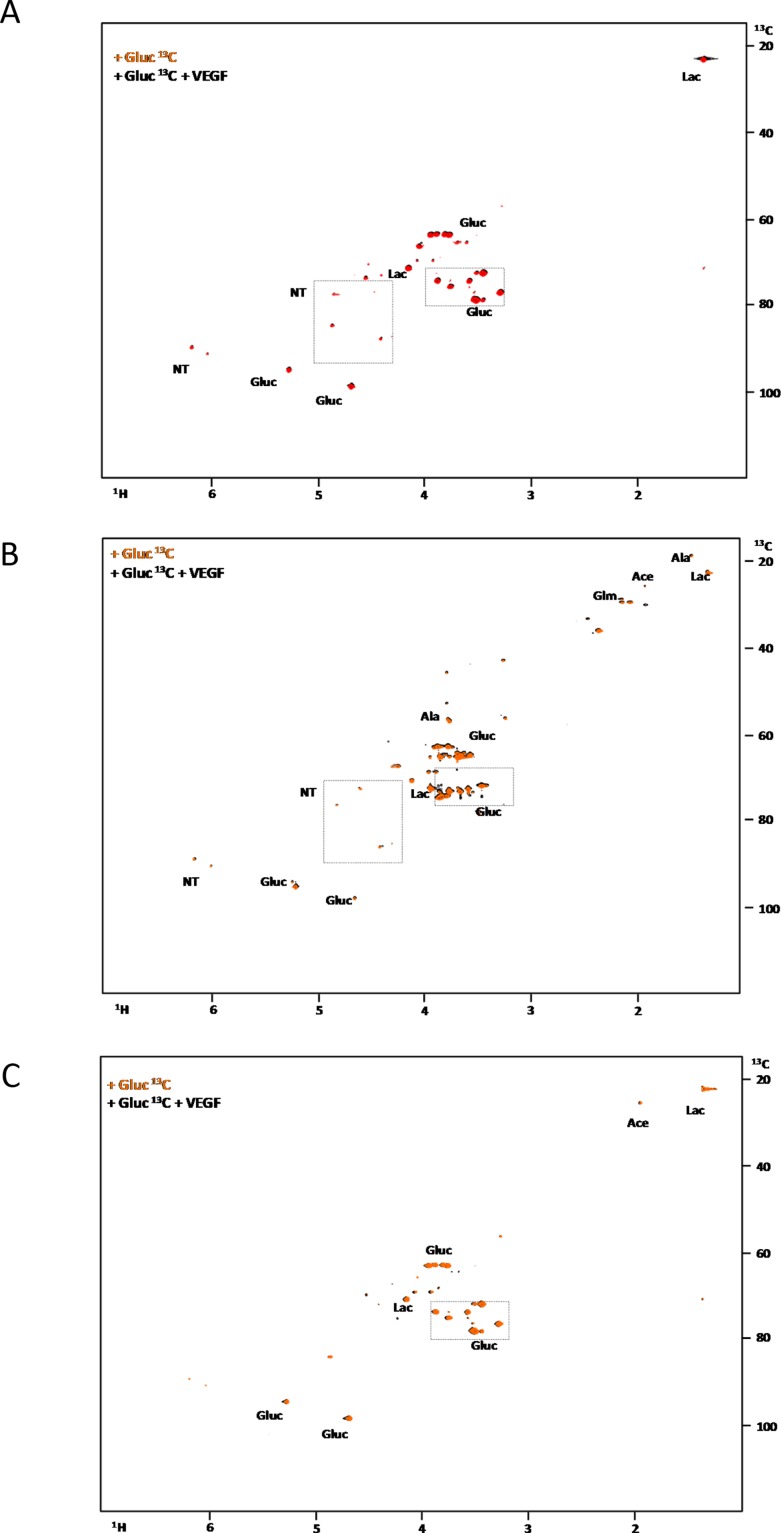

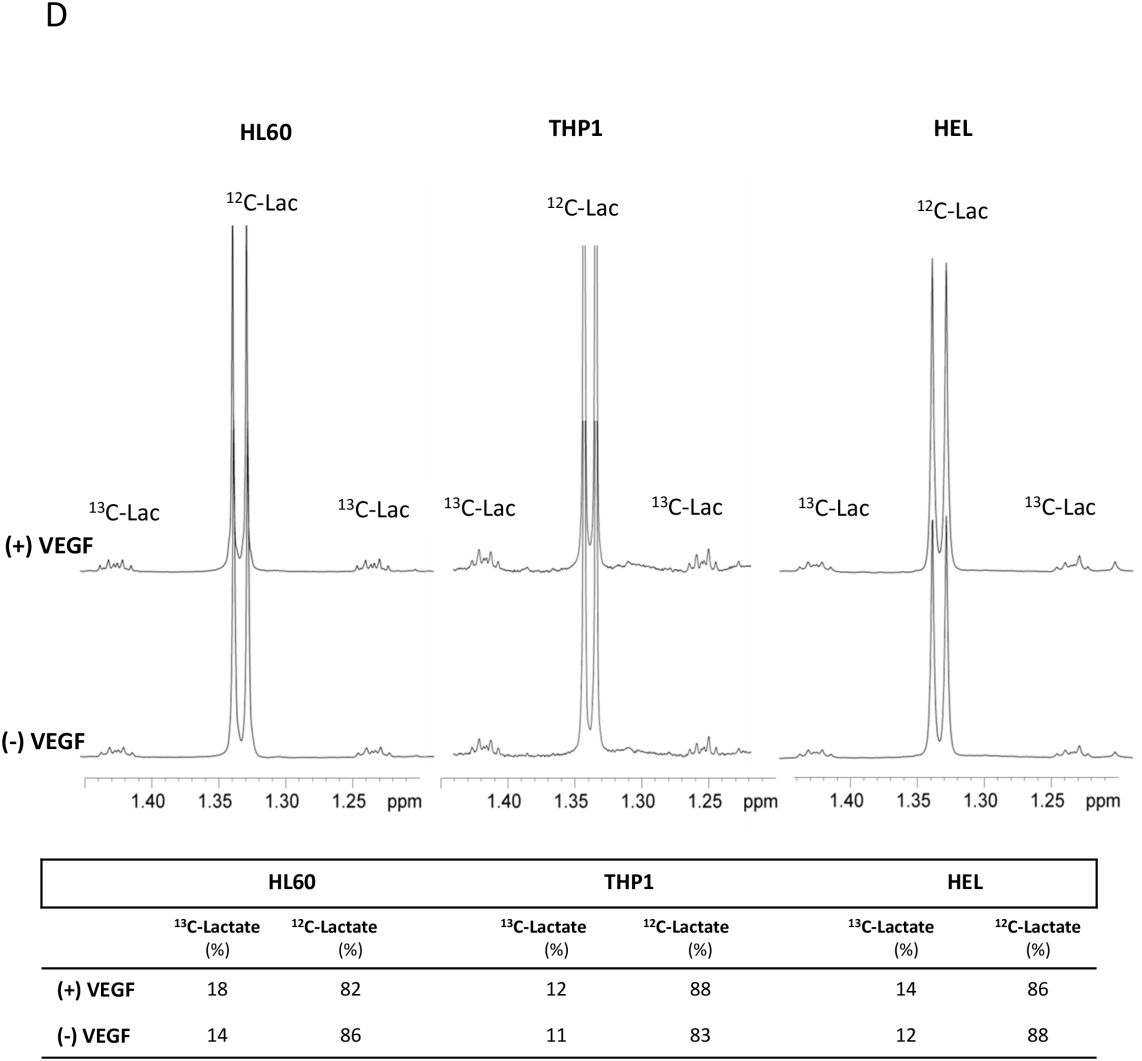
The effect of VEGF in glucose metabolism in AML cell lines ^1^H-^13^C-HSQC NMR spectra of HL60 **(A)**, THP1 **(B)** and HEL **(C)** intracellular extracts cultured with ^13^C-U-glucose in the absence and in the presence of 10μg/mL of VEGF. **(D)** ^1^H-NMR spectra highlight of the lactate methyl group when the three cell lines (HL60, THP1 and HEL) were cultured with ^13^C-U-glucose: in the absence (spectra below) and in the presence of VEGF (spectra above). The percentage of ^13^C-lactate and ^12^C-lactate present each condition is indicated in the board. Gluc- glucose; Ace- acetate; Glm- glutamine; Lac- lactate and NT- ribosyl moiety of nucleotides. Results were obtained from 3 independent replicates, and representative figures are presented.

In order to follow the glucose metabolism, ^13^C glucose was used as a carbon source and analysed by NMR spectroscopy. In HL60 and THP1, glucose was preferentially used to produce lactate (through glycolysis) and sugar pentose rings in nucleotides (Figure 2A and 2B). Again, the production of nucleotides was increased in the presence of VEGF (Figure 2A and 2B). In HEL cell line, glucose was used to produce lactate and acetic acid (TCA cycle intermediate) independently of VEGF presence (Figure 2C).

The *ratio* of ^13^C and ^12^C in the intracellular lactate, increased from 14% to 18%, when ^13^C-glucose is used in the presence of VEGF in the HL60 cells. Whereas in the other cell lines, this *ratio* was almost constant (Figure 2D).

NMR revealed that lactate and glucose metabolism is modulated by VEGF in HL60 (promyelocytic) and THP1 (monocytic) cell lines but not in the erythroblastic cell line HEL.

### Expression of monocarboxylate transporter 1 (MCT1) is regulated by VEGF and MCT4 is regulated by lactate

**Figure 3:**
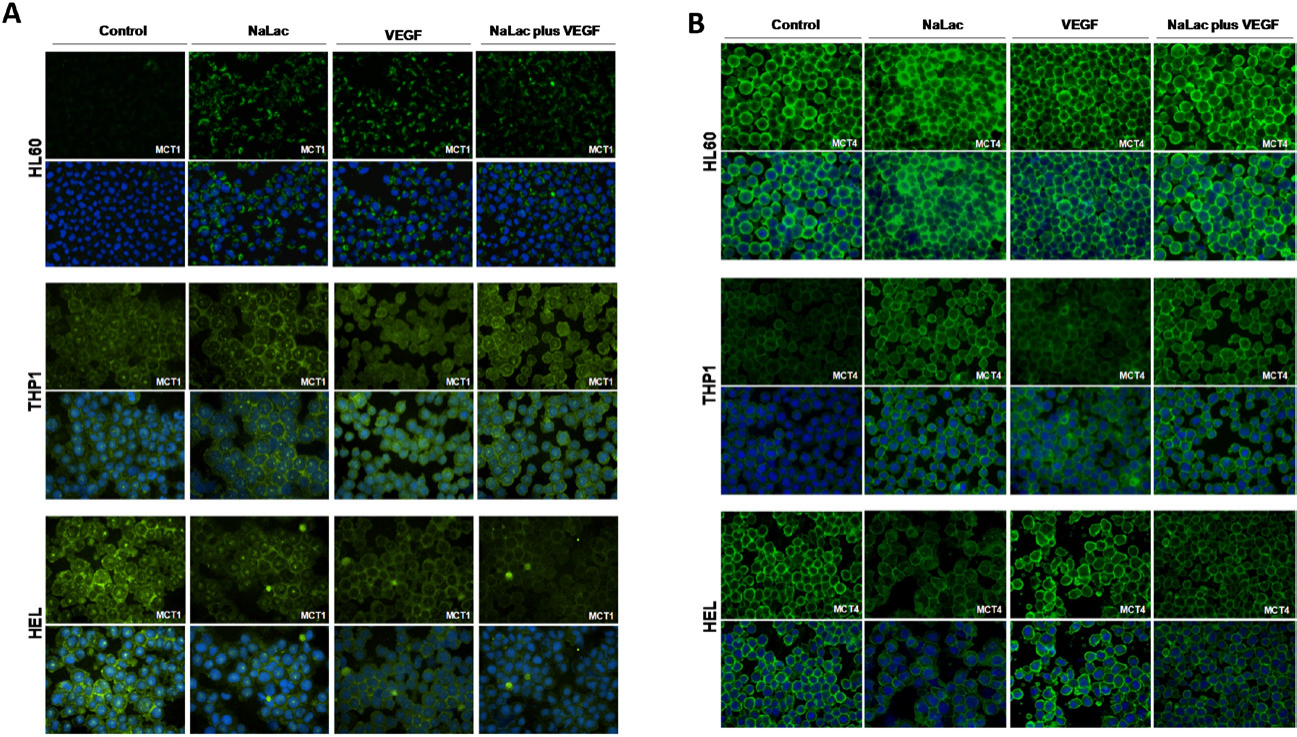

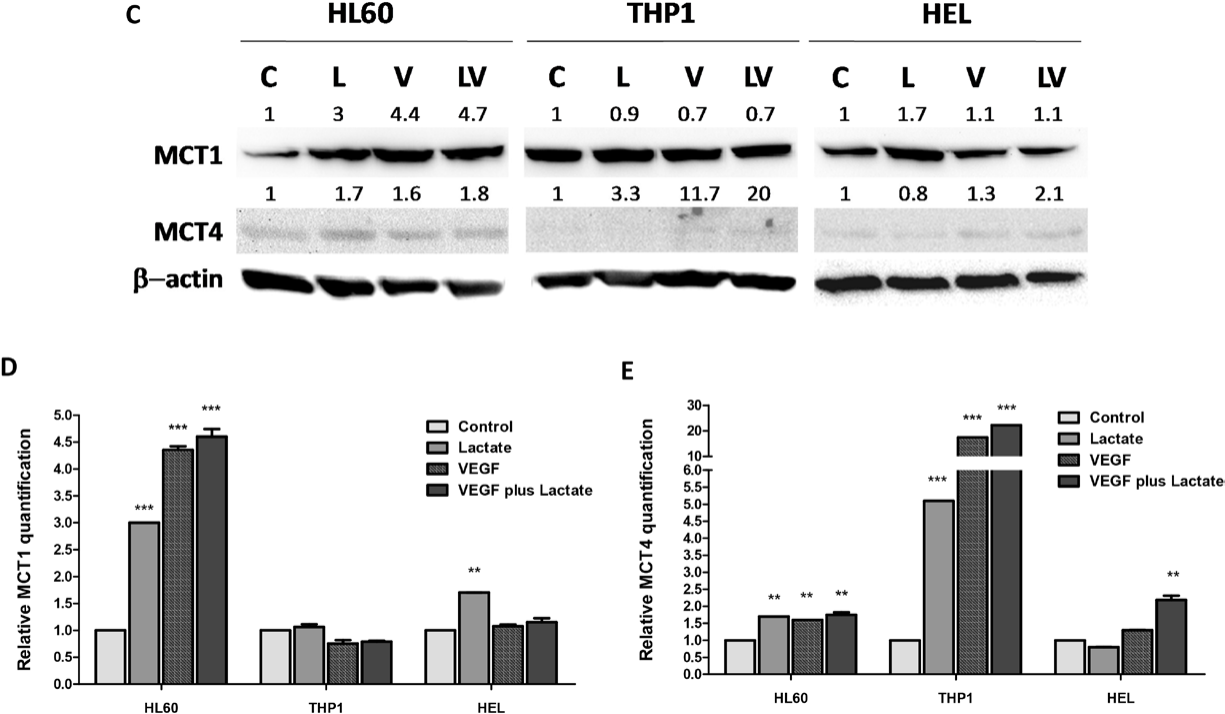

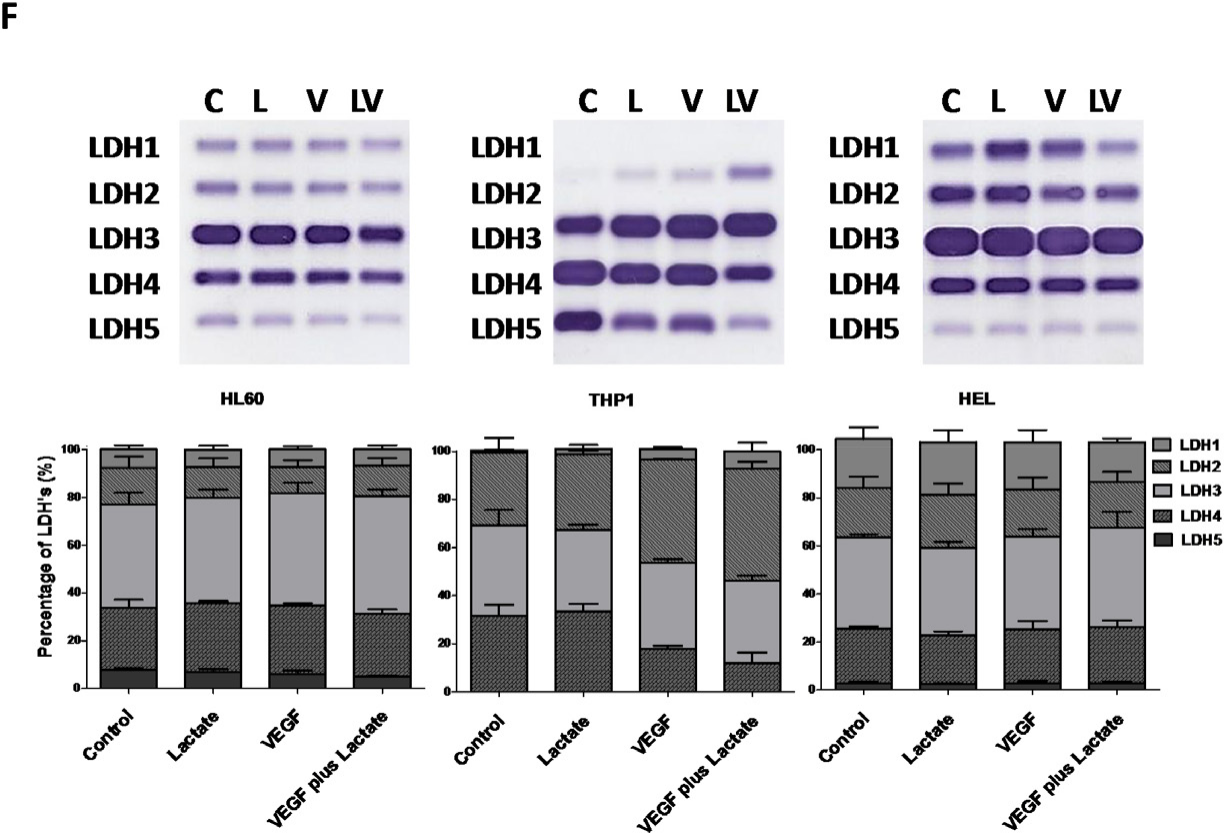
Expression of MCT1, MCT4 and LDH isoenzymes under lactate and VEGF stimuli Immunofluorescense and western blotting was performed in order to evaluate the effect of lactate and VEGF in the expression of MCT1 and MCT4, in HL60, THP1 and HEL cell lines. Immunofluorescense for the detection of MCT1 **(A)** and MCT4 **(B)**, western bloting for MCT1 and MCT4 **(C)** which were respectively quantified **(D** and **E)** using control conditions of each cell line after normalization for β-actin and **(F)** evaluation of LDH isoenzymes in an agarose gel electrophoresis (LDH Isoenzymes Electrophoresis Kit; SRE612K, Interlab) and bands quantification in an EasyFix Interlab G26 equipment. C-Control, L-Lactate, V-VEGF, LV-NaLac plus VEGF. Error bars represent standard deviation; statistical significance **p<0.01, ***p<0.001. Results were obtained from 3 independent replicates, and representative figures are presented.

Monocarboxylate transporters are essential for lactate import and export. In cancer context MCT1 is described as being expressed in cells that preferentially import and consume lactate whereas MCT4 is more prone to export lactate [12]. Although a report in glycolytic cells from brain tumors has described MCT1 as a mediator of lactate export [18]. By immunofluorescense and western blotting, it was seen that the levels of MCT1 were increased after lactate and VEGF exposure in HL60. MCT1 in THP1 cells remains unchanged upon all culture conditions. In HEL cell line, although immunofluorescense showed a decrease in MCT1 with lactate and VEGF, by western blotting it was observed an increase with lactate in the absence of VEGF (Figure 3A, 3C and 3D). Regarding MCT4 expression, lactate and VEGF increase its expression in HL60 and THP1, whereas only VEGF increases MCT4 expression in HEL cell line (Figure 3B, 3C and 3E). Despite some differences in the basal levels of MCT1 and MCT4, all cell lines express both transporters (Figure 3A, 3B and 3C).

Overall, the expression of MCTs in all cell lines is not limiting for the import and export of lactate in order to support respectively the lactate and glucose consumption. Moreover, there is an adjustment of MCT1 expression in order to accomplish the metabolic adaptation in the presence of VEGF in HL60 and THP1 and upon the supplementation with lactate in HEL.

### AML cell lines express a panel of lactate dehydrogenases (LDH) isoenzymes capable of synthesizing and degrading lactate

LDH isoenzymes have different specificities and kinetics. LDH5 isoenzyme preferentially converts pyruvate into lactate and LDH1 preferentially converts lactate into pyruvate. The LDH 2, 3 and 4 isoenzymes have intermediate affinities to catalyse the two way reaction [11]. By agarose gel electrophoresis, it was observed that all isoforms were expressed in HL60 and HEL cells, and their expression was not modulated by neither VEGF nor lactate (Figure 3F). In THP1 cell line, LDH1 was not detected, whereas LDH2 increases with VEGF and lactate exposure and the opposite occurs for LDH5 (Figure 3F). Nevertheless, the most abundant isoform in all cell lines was LDH3, which is able to catalyse the two way lactate/pyruvate reaction, allowing the consumption and the production of lactate.

Therefore, the expression of LDHs in all cell lines is not limiting for the metabolic fitness to lactate and glucose consumption

These results, together with the increased production of nucleotides in HL60 and THP1 cell lines with VEGF (Figure 1), prompted us to assess if different metabolic demands were directly related to cell proliferation and cell cycle status, disclosing the role of VEGF in metabolic fitness.

### VEGF interferes differently with cell proliferation of HL60 and HEL cell lines

**Figure 4:**
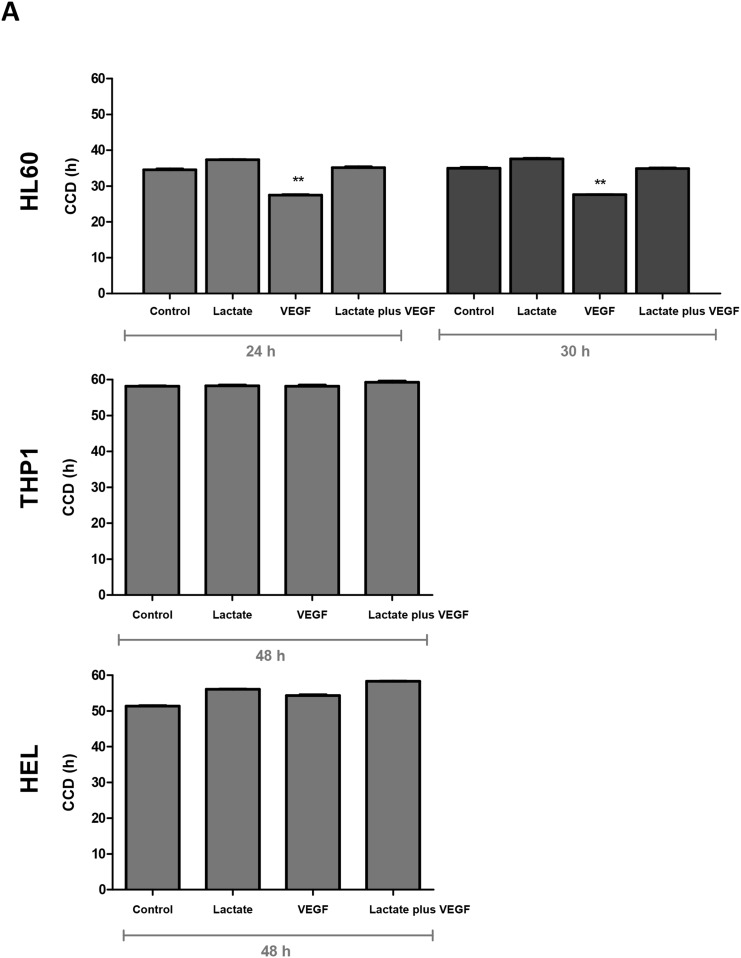

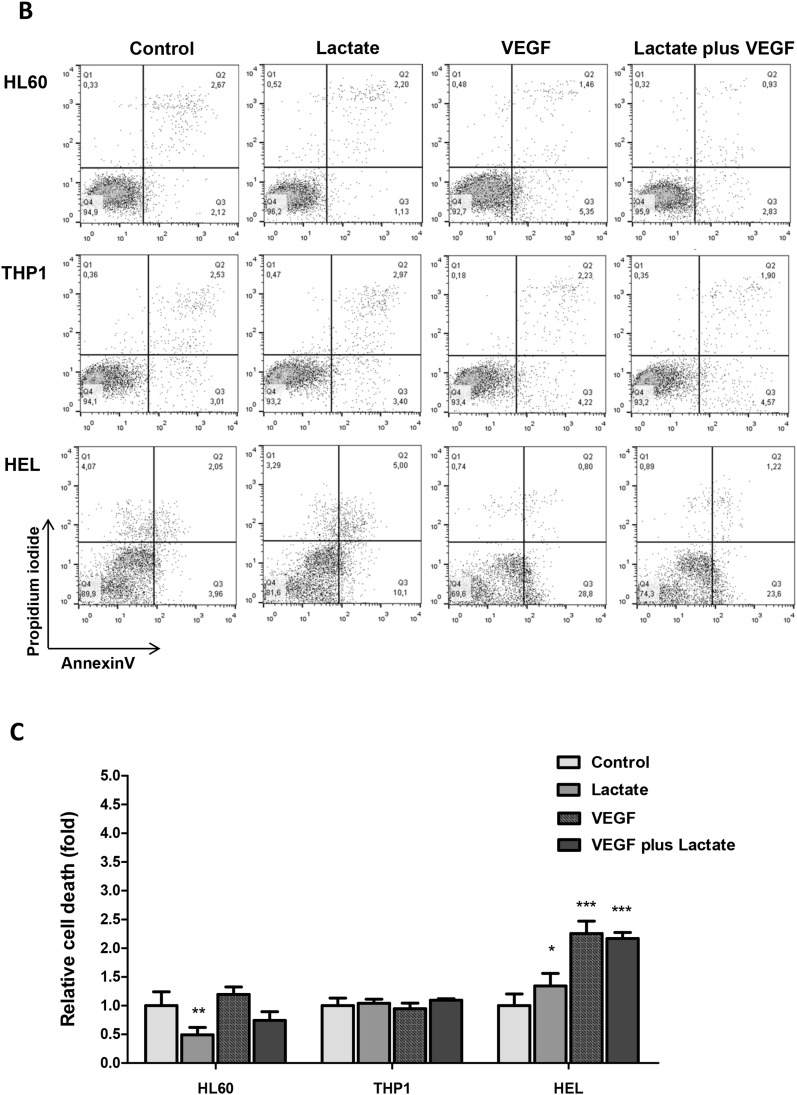
The effect of lactate and VEGF in cell cycle and cell viability in AML cell lines Cell cycle duration (CCD) was determined by flow cytometry in ethanol fixed cells stained with propidium iodide (PI) at duplication time (DT) of HL60, THP1 and HEL cell lines **(A)**. Cell death was evaluated by flow cytometry in annexin V and PI double stained cells: **(B)** flow cytometry plots, and **(C)** relative cell death quantification. Error bars represent standard deviation; statistical significance *p<0.1, **p<0.01, ***p<0.001. Results were obtained from 6 independent replicates, and representative histograms are presented.

The increased production of nucleotides in HL60 and THP1 cell lines, detected by NMR, in the presence of VEGF was a sign that VEGF might stimulate cell proliferation. So, proliferation curves were established in order to evaluate the influence of lactate and VEGF in cell population doubling time (DT) and in cell cycle duration (CCD). It was observed that VEGF significantly decreases the DT (p<0.01) in HL60 and lactate does not interfere with DT (Table 1). DT of THP1 cell line is not influenced neither by VEGF nor lactate (Table 1). But, HEL cell line exposed to both lactate and VEGF showed a significant increase in the DT (p<0.05) (Table 1). CCD was calculated for HL60 at 30h and for THP1 and HEL cell lines at 48h of cell culture, adjusted to the DT determined for each cell line at each conditions (without lactate and VEGF) (Table 1). In HL60, CCD decreased with VEGF exposure (p<0.001) (Figure 4A). In THP1, no difference in CCD was observed upon exposure to VEGF or lactate. In contrast, exposure to VEGF and lactate, in separate and in combination, increased CCD slightly in the HEL cell line (Figure 4A).

**Table 1:** Determination of the doubling time (h) of each cell line in control conditions and exposed to lactate (10mM), VEGF (10ng/ml) and lactate plus VEGF

Cell Line	Control (h)	Lactate (h)	VEGF (h)	Lactate plus VEGF (h)
**HL60**	29.2	30.8	22.9**	28.8
**THP1**	48.1	47.8	48.4	50.1
**HEL**	40.5	43.4*	44.2*	47.4**

Statistical significance: *p<0.05; ** p<0.01.

These assays showed the VEGF effect in mitogenesis varies according to the AML cell line, being favourable to promyelocytic cell line (HL60), unfavourable to erythroblastic cell line (HEL) and indifferent to monocytic cell line (THP1).

### The promyelocytic (HL60) and the monocytic (THP1) cell lines are fitted to lactate and VEGF

Since the dynamics of a cell culture depends on cell proliferation and cell survival, the determination of cell death in all culture conditions was mandatory.

The relative cell death quantification showed that HL60 cell line died less when cultured with lactate (p<0.01), whereas VEGF did not interfere with cell survival; THP1 survival was not affected neither by the presence of lactate nor VEGF, and HEL showed a lower cell viability (higher cell death) in the presence of lactate (p<0.05) and VEGF (p<0.001) in separate and in combination (p<0.001) (Figure 4B and 4C).

Again, it was shown that the promyelocytic (HL60) and the monocytic (THP1) cell lines are very well adapted to a microenvironment rich in lactate and VEGF, being favourable for HL60 and indifferent to THP1, in terms of cell death. Both lactate and VEGF are harmful to erythroblastic cell line (HEL).

### Expression and localization of MCT1 and MCT4 in BM from AML patients

**Figure 5:**
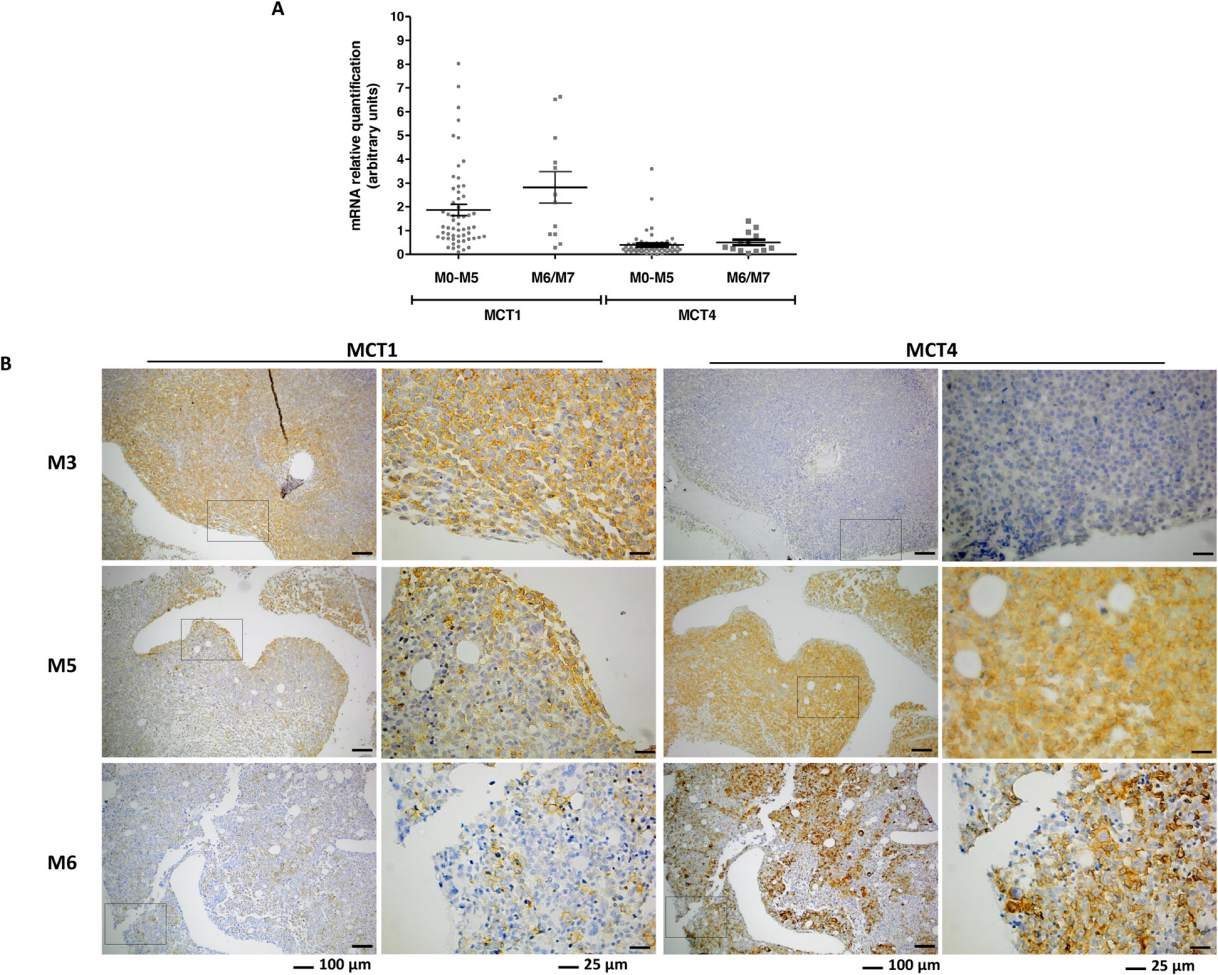
Expression of MCT1 and MCT4 expression in bone marrow (BM) samples from AML patients MCT1 and MCT4 mRNA was quantified by relative quantifying real-time PCR, using as reference a pool of mRNA of disease free individuals **(A)** and MCT1 protein was evaluated by immunohistochemistry in paraffin embedded section of BM **(B)** images were acquired in magnification of 100x and 400x.

The expression of MCT1 in BM specimens of AML patients from M0-M5 and M6/M7 groups is significantly higher than MCT4 expression (p<0.0001) (Figure 5A). However, there was no difference in the expression levels of mRNA from MCT1 and MCT4 between the two groups of patients, M0-M5 and M6/M7 (Figure 5A).

The localization of MCT1 and MCT4 was also assessed in BM biopsies. MCT1 positive cells localized differently between the two groups of patients, being associated with a paratrabecular localization in M0-M5 (p=0.003) and diffuse/interstitial in M6/M7 (p=0.017) (Figure 5B and Table 2). MCT4 positive cells were associated with a central localization in M0-M5 patients (p=0.022), whereas in M6/M7 patients there was no preferential localization in BM (Figure 5B and Table 2). The fact that the p value for the localization of MCT1 and MCT4 between M0-M5 and disease free individuals is close to 1 means that the distribution pattern of MCT1 and MCT4 positive cells is very similar to the normal pattern in BM (Table 2).

**Table 2:** MCT1 and MCT4 cell localization in bone marrow (BM) of AML patients with M0- M5 (n=28) and M6/M7 (n=7) subypes

	AML (M classification)	Cells localization in BM								NE
		Para-trabecular	*p* value	Central	*p* value	Diffuse	*p* value	Negative	*p* value	
**MCT1**	**M0-M5**	64.3% (18/28)	0.003	3.6% (1/28)	0.464	21.4% (6/28)	0.017	0% (0/28)	-	10.7% (3/28)
	**M6/M7**	0% (0/7)		14.3% (1/7)		71.4% (5/7)		0% (0/7)		14.3% (1/7)
	**Disease free BM**	60% (3/5)	0.840 ^a^ 0.073 ^b^	0% (0/5)	0.935 ^a^ 0.473 ^b^	40% (2/5)	0.747 ^a^ 0.259 ^b^	0% (0/5)	-	0% (0/5)
**MCT4**	**M0-M5**	17.9% (5/28)	0.256	14.3% (4/28)	0.022	53.6% (15/28)	0.550	7.1% (2/28)	0.748	7.1% (2/28)
	**M6/M7**	0% (0/7)		57.1% (4/7)		28.6% (2/7)		0% (0/7)		14.3% (1/7)
	**Disease free BM**	20% (1/5)	0.999 ^a^ 0.659 ^b^	20% (1/5)	0.970 ^a^ 0.150 ^b^	60% (3/5)	0.995 ^a^ 0.668 ^b^	0% (0/5)	0.779 ^a^ 1 ^b^	0% (0/5)

AML patient subtypes were classified according to WHO classification. Disease free BM (n=5) were used as control. NE - not evaluated.

a) *p* value of M0-M5 vs Disease free BM; b) *p* value of M6/M7 vs Disease free BM.

In M0-M5 specimens, MCT1 positive cells are mainly immature aberrant cells, which may be leukemia blasts, whereas MCT4 is expressed also in normal mature and immature BM cells. In M6/M7 specimens, MCT1 and MCT4 are expressed in all cell types.

### MCT1 can be used as a vehicle for the delivery of cytotoxic monocarboxylates, as bromopyruvic acid (BPA)

**Figure 6:**
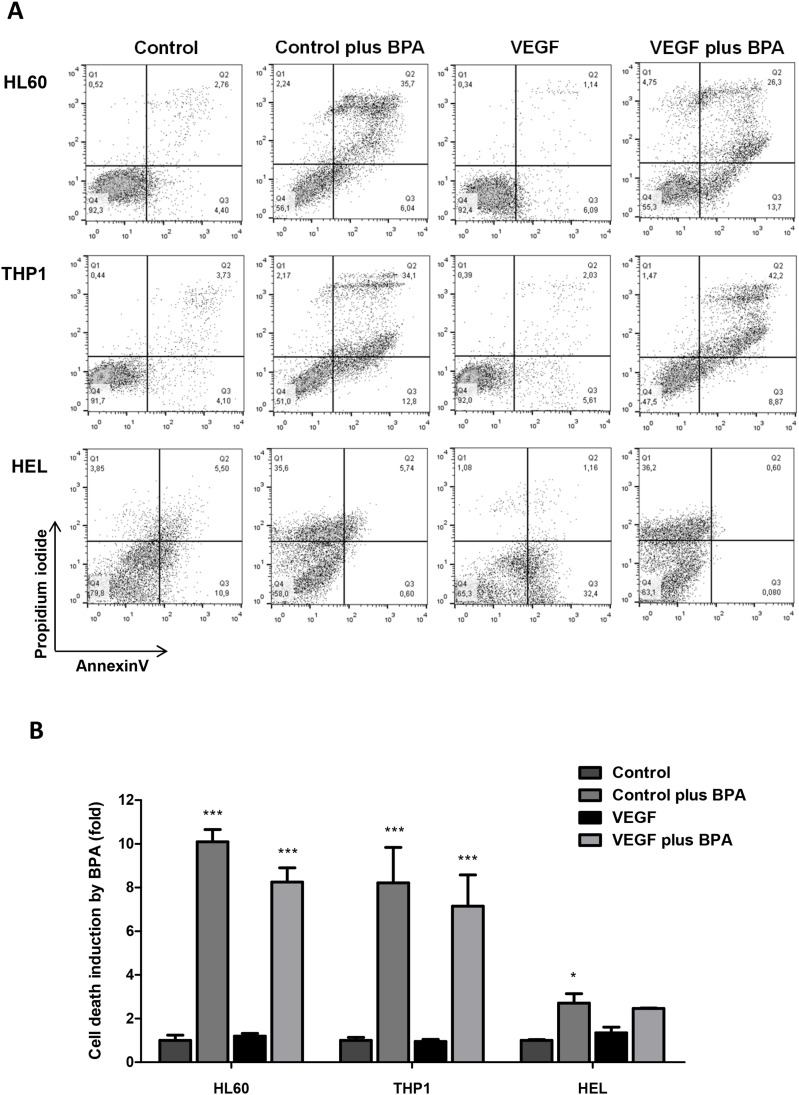
The effect of bromopyruvic acid (BPA) in cell viability in AML cells with and without VEGF stimulus Cell death was evaluated by flow cytometry in annexin V and PI double stained cells. HL60, THP1 and HEL cells were exposed to BPA (LC50) in the presence or absence of VEGF: **(A)** flow cytometry plots, and **(B)** relative cell death quantification. Error bars represent standard deviation; statistical significance *p<0.1, ***p<0.001. Results were obtained from 6 independent replicates, and representative histograms are presented.

In order to verify if MCT1 can be used as a vehicle to uptake cytotoxic monocarboxylates, we tested the effect of bromopyruvic acid (BPA) in AML cells viability. The lethal concentration 50 (LC50) of BPA was proportional to the levels of MCT1 expression in each cell line (Figure 3A, 3B and 3C and Table 3). HEL, which was the cell line that expressed higher levels of MCT1 showed a LC50= 0.026mM for BPA, whereas HL60 and THP1, which expressed similar levels of MCT1 (but lower than HEL), showed a LC50= 0.053mM and LC50= 0.045mM respectively (Figure 6A and 6B and Table 3).

**Table 3:** Determination of the lethal concentration 50 (LC50) of bromopyruvic acid (BPA) in HL60, THP1 and HEL AML cell lines

Cell Line	LC50 BPA (mM)	95% confidence intervals	R^2^
**HL60**	0.053	0.047 to 0.059	0.9272
**THP1**	0.045	0.0424 to 0.047	0.9580
**HEL**	0.026	0.021 to 0.028	0.8604

LC50: Lethal concentration of BPA where 50% cell death were observed.

95% confidence intervals for the LC50 values.

R^2^: coefficient of determination.

When exposed to BPA, all AML cell lines showed statistically significant higher levels of cell death, comparing with control conditions (Figure 6A and 6B). The same results were verified in HL60 and THP1 cells exposed to BPA in combination with VEGF (Figure 6A and 6B). However, there was no difference in cell death levels between cells exposed with BPA and BPA plus VEGF in all cell lines (Figure 6A and 6B). From the 3 cell lines, HEL cells showed the lower fold induction of cell death induced by BPA in control conditions and with VEGF. In the presence of VEGF the cell death induced by BPA was not significant (Figure 6A and 6B). So, it seemed that cell death was due to BPA cytotoxic action and VEGF did not potentiate this effect.

## DISCUSSION

Our work showed for the first time that the metabolic adaptation of AML cells, depending on the lineage, is beneficial for leukemia cells to survive in bone marrow (BM) microenvironment. Cell lines from different AML subtypes behave differently upon VEGF stimuli, being its effect reflected in the way cells proliferate and survive with a concomitant metabolic adaptation. As we will further discuss, plasticity seems to be a strength of HL60 promyelocytic cell line as it can adapt to different metabolic conditions; THP1 monocytic cell line seems to be well adapted to the presence of lactate and VEGF, and HEL erythroid cells are misfit to lactate and VEGF rich microenvironment.

In AML cells from promyelocytic (M3) and monocytic (M5) lineages, we saw that glucose was used mainly to sustain cell proliferation probably through Pentose Phosphate Pathway (PPP), as seen HL60 (promyelocytic) and THP1 (monocytic) cells incorporate carbons from ^13^C-glucose in nucleotides, in the presence of VEGF (Figure 2A and 2B). HEL cells did not use glucose to produce nucleotides (Figure 2C). The quantification of ^13^C-lactate (supplemented in the media) and ^12^C-lactate (produced from glucose) in the cells (Figure 1D and 2D) showed that VEGF increases glycolysis rate in HL60 but not in THP1 and HEL. Since the *ratio* of ^12^C-lactate and ^13^C-lactate increased in HL60 upon VEGF exposure and remained the same in THP1 and HEL in the presence and absence of VEGF. However, in HL60 and THP1 the incorporation of ^13^C from glucose in pentoses of nucleotides moieties upon VEGF exposure indicates that VEGF promotes a switch of glycolysis intermediates towards nucleotides synthesis, possibly through PPP. (Figure 2A and 2B) Increased rates of PPP is a phenomenon that was recently described in sepsis, when myeloid cells are engaged with proliferation and differentiation [19] and also in cancer cells [20, 21] including leukemia [22].

The influence of VEGF in the HL60 metabolic switch of lactate and glucose can be related to cell proliferation as HL60 had a lower duplication time (DT) and cell cycle duration (CCD) in the presence of VEGF in comparison to cells cultured in control conditions and in the presence of lactate (Table 1, Figure 4A). Accordingly, several studies have shown that glycolysis rate increases in highly proliferative cells [21, 23–25] and PI3K/Akt/mTOR pathway is relevant in glycolysis regulation [25, 26]. In addition the existence of an autocrine loop of VEGF with its receptor 2 (VEGFR2/KDR) in HL60 cells is well known [27] and interestingly VEGFR2 can activate PI3K/AkT/mTOR pathway pathway [28, 29]. HL60 cultured in the presence of combined lactate and VEGF had a DT similar to control conditions (Table 1), maybe because HL60 need to adapt to lactate bioavailability and VEGF can be the modulator of this metabolic switch through the stimulation of an increased proliferation rate. In the end, lactate bioavailability is advantageous for these leukemia cells, because HL60 cell death decreases in the presence of lactate (Figure 4B and 4C). THP1 (monocytic) did not show any alteration in CCD, DT and cell death in the presence of lactate or VEGF (Table 1 and Figure 4A, 4B and 4C), denoting a quite good fitness to those conditions. THP1 cell line are from monocytic lineage and it was proven to differentiate, *in vitro*, into macrophages [30–32]. Some authors, showed the process of monocytes differentiation involves several metabolic adaptations and lactate consumption is one of them [33–35]. PI3K/Akt/mTOR pathway plays a role in metabolic adaptation associated to macrophages differentiation [36]. The increased DT and cell death levels in HEL cells due to lactate and VEGF indicates this cell line is not adapted to lactate consume and VEGF has no effect in regulating lactate metabolism (Table 1 and Figure 4A, 4B and 4C).

As mentioned, HL60 and THP1 cell lines were really well adapted to lactate and they were able to use ^13^C from lactate to synthesize several amino acids, TCA cycle intermediates and nitrogen bases from nucleotides (Figure 1A and 1B). TCA cycle is the main aneuplerotic center in eukaryotic cells, supplying several other biomass producing metabolic pathways [37]. Evidently HEL (erythroid) cells were not well adapted to the use of lactate, since they only produce succinate and acetate that are directly converted from ^13^C-lactate, (Figure 1C). HEL cells used ^13^C-glucose and ^13^C-lactate to produce almost the same pattern of organic compounds (Figure 1C and 2C). Recently, Xu et al (2016) [38] showed that erythroblastic cells have the glucose metabolism regulated by a panel of genes commanded by HIF2α, and those genes are preferentially expressed under hypoxia. In our study, we did not tested metabolic adaptation in hypoxic conditions but it is an interesting subject that we will address in the future.

Metabolic adaptation involves an orchestrated remodelling of metabolic intervenients and in lactate metabolism the most important elements are monocarboxylate transporters (MCTs) [9, 10] - in cancer MCT1 and MCT4 seem to be the most relevant [11, 12, 39]; and lactate dehydrogenases (LDH), which are able to convert lactate and pyruvate in both ways and have even been considered as putative therapeutic targets in cancer [40, 41]. HL60 reacted to lactate and VEGF by increasing MCT1 levels and THP1 expresses high levels of MCT1, which are maintained upon lactate and VEGF exposure (Figure 3A and 3C). So MCT1 must be pivotal in lactate uptake by cancer cells (as HL60 and THP1) that are able to fully metabolize lactate as described in other cancer models [12, 39]. In addition, this is the first time that VEGF is described as a regulator of MCT1 expression. Another element of VEGF family, VEGF-C, has been described as a regulator of MCT4 in cervical adenocarcinomas [42]. As seen, the panel of LDHs expressed was not limiting for metabolic fitness, as LDH3 is the prevalent isoform in all cell lines cultured in all conditions and it is capable of catalysing the interconversion of lactate into pyruvate [11].

**Figure 7:**
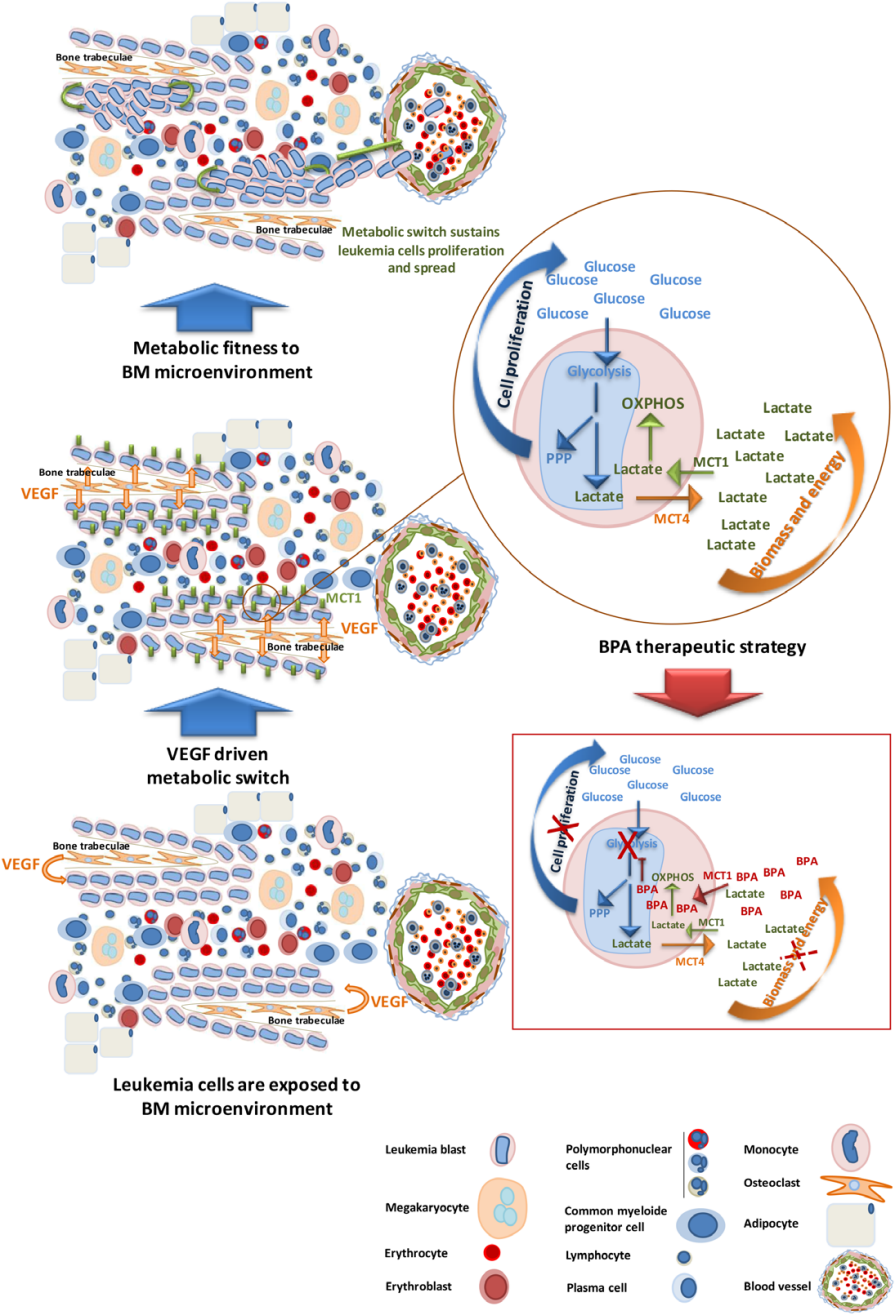
AML subsets benefit from BM microenvironment to proliferate and carry on disease progression- bromopyruvic acid (BPA) metabolic therapeutic strategy M0-M5 AML cells localize preferentially near the bones (paratrabecular homing) taking advantage of the VEGF modulating gradient. VEGF stimulates AML cells proliferation and regulates the expression of MCT1, contributing for the metabolic fitness of cells to the microenvironment. Proliferating cells increase the uptake of glucose for glycolysis to sustain mainly pentose phosphate pathway (PPP) crucial for cell division; lactate resulting from glycolysis must be secreted, through MCT4, in order to maintain internal pH. The uptake of this lactate to sustain oxidative phosphorylation (OXPHOS) is mediated by MCT1. Cancer cells fitted to BM metabolic microenvironment will carry on AML progression and systemic diffuse. Bromopyruvic acid (BPA) can be used as a metabolic drug, transported mainly through MCT1. As a glycolysis inhibitor, BPA will abrogate glycolysis disturbing PPP and cell proliferation, and also will partially disturb OXPHOS because once glycolysis is abrogated the levels of lactate available to be used in OXPHOS will drastically decrease. Hence, BPA will interfere with biomass and energy production, contributing for decreased cell viability and disease constraining.

In the context of BM microenvironment, leukemia cells of promyelocytic and monocytic lineages can take advantage on the high levels of VEGF near the bone trabeculae, where it is produced by osteoclasts [43–45]. This potentiates the metabolic switch as we described here for the first time, VEGF regulates the expression of MCT1, allowing internalization of lactate from glycolysis to be used in the TCA cycle and OXPHOS for energy and biomass production (Figure 7).

This hypothesis is corroborated by the analysis of BM samples from patients with AML M0-M5, showing that MCT1 positive cells prefer to localize near the bones (paratrabecular region) (Figure 5B and Table 2). In contrast, in AML M6/M7 BM samples MCT1 and/or MCT4 positive cells are mainly dispersed without preferential location in BM (Figure 5B and Table 2). In the normal like/disease free BM analyzed, we observed that MCT1 positive cells also localized near the bone trabeculae and MCT4 positive cells are diffuse in BM, reinforcing that metabolic adaptation allows AML M0-M5 to benefit from a natural BM organization driven also by VEGF, as it is already described [27, 46–49]. The metabolic symbiosis is possibly working on BM not only among leukemia cells but also between leukemia cells and normal cells [8, 50, 51].

We tried to take advantage on the ability of AML cells to uptake monocarboxylates to induce cancer cells death by using bromopyruvic acid (BPA). BPA is a relevant anticancer drug *in vitro*, tested in animal models [52–55] and in humans [56]. In our study, BPA lethal capacity was shown to be related to the levels of MCT1 expression (Figure 3A, 3C and 6A and Table 3). MCT1 was described as a BPA effective transporter in other studies [57, 58] and MCT1 and other MCTs have been considered suitable vehicles for drug delivery [9, 59, 60]. Comparing to HL60 and THP1, HEL cells had a lower fold change in relative cell death induced by BPA both in the presence and absence of VEGF (Figure 6B). Again, HL60 and THP1 proved to be more competent in monocarboxylates uptake than HEL, since HL60 and THP1 showed a cell death upon BPA exposure 10 fold higher than without BPA, with or without VEGF (Figure 6B). In addition, BPA is more death effective in cells that have high glucose demands, namely cancer cells that proliferate more [61]. The cell death profile was also different between cell lines, as it is described for BPA in other cancer models [62]: HL60 and THP1 showed double PI and annexin V positive cells, consistent with late apoptosis or necroptosis, whereas HEL cells died mainly through necrosis exhibited by the high PI positivity (Figure 6A). Beyond its cytotoxic effect as an inhibitor of glycolysis [63] and consequently TCA cycle [64], BPA has been reported as a sensitizer of cancer cells to conventional chemotherapy, since as an alkylating agent, it reacts with glutathione [52]. As mentioned in several studies, high levels of glutathione in cancer cells is a mechanism of resistance to drugs [65–67]. However in a case report it was described that if the levels of glutathione are too high it also abrogates BPA action, being indicated the intake of paracetamol to deplete glutathione [68].

Further studies will be developed in order to find which signaling pathways are activated by VEGF to modulate MCT1 expression. We believe that is VEGF as a tumor growth factor [27, 69, 70] that is acting on the metabolic remodeling of AML cells and MCT1 expression is a part of these phenotypic changes. In some cancer models mTOR is pointed out as a regulator of MCT1 expression [71] and PI3K/Akt/mTOR pathway is also a regulator of VEGF expression [72, 73]. Moreover, the VEGF receptor 2 (VEGFR2/KDR) is involved in the activation of PI3K/Akt/mTOR pathway [28, 29]. It is also known that VEGF:VEGFR2 can work in autocrine loops in several cancer models [27, 74–76], including refractory leukemia [77]. So, the scenario of a continuous metabolic loop, in which VEGF acts on VEGFR2 to activate PI3K/Akt/mTOR pathway that will induce the expression of both MCT1, driving the metabolic fitness, and VEGF that will perpetuate the metabolic adaptation of leukemia cells, can be a possibility. On the other hand, succinate which results directly from lactate is also described as a modulator of VEGF expression [78], contributing to sustain the metabolic loop.

Our study, besides opening several different research avenues, it also shed one more light on the way metabolic route can be used to disturb and kill cancer cells. In this case, MCT1 as a transporter of toxic monocarboxylates, as BPA, can be a good ally (Figure 7) to treat patients with MCT1 positive AML.

## MATERIALS AND METHODS

### Cell culture conditions

Cell lines derived from patients with acute promyelocytic leukemia (M3) (HL60: ATCC^®^ CCL-240 ™), acute monocytic leukemia (M5) (THP1: ATCC^®^ TIB-202™) and acute erythroid leukemia (M6) (HEL: ATCC^®^ TIB-180™) were obtained from American Type Culture Collection (ATCC). Cells were maintained at 37°C in a humidified 5% CO_2_ atmosphere in RPMI 1640 medium (31870, Gibco - Life Technologies Inc) supplemented with 4 mM L-glutamine (250330-81, Invitrogen™, Life Technologies Inc), 10% Fetal bovine serum (FBS) (S 0615, Invitrogen™, Life Technologies Inc) and 1% Antibiotic-Antimycotic (15240062, Invitrogen™, Life Technologies Inc). Before any *in vitro* experiment, cells were synchronized under starvation (culture medium without FBS), overnight at 37°C and 5% CO_2_.

Experimental conditions for nuclear magnetic resonance (NMR) spectroscopy assays included 5x10^7^cells exposed to 20% of ^13^C compounds (^13^C-[U]-lactate- 660817, Sigma-Aldrich^®^, ^13^C-[U]-glucose- 389374, Sigma-Aldrich^®^), to a final concentration of 10 mM lactate (NaLac) and 20 mM D-glucose with/without 10 ng/mL of vascular endothelial growth factor (VEGF) (V7259, Sigma-Aldrich^®^) plus 10U/mL heparin (H3149, Sigma-Aldrich^®^) in 40 mL of RPMI 1640 medium, supplemented with 4 mM L-glutamine 1% FBS, 1% AA, for 24h. Supernatants were collected and stored at - 80°C.

For the proliferation assay and immunofluorescence 1x10^6^ cells were seeded in 12-well plates; for S and G2-M cell cycle phase duration and cell death analysis cells (5x10^4^ cells) were seeded in 48-well plate; and for LDHs determination 2.5x10^5^ cells were seeded in 24-well plate and maintained in control conditions and/or 10 mM lactate and/or 10 ng/mL VEGF plus 10 U/mL heparin. For cell death analysis, cells were additionally exposed to bromopiruvic acid (BPA) (16490, Sigma-Aldrich^®^) for 24h in a range of 0.01 mM – 30 mM, in order to determine the lethal dose 50% (LC50). After that for each cell line, cells were exposed to LC50 both in control and under VEGF supplementation.

### Nuclear magnetic resonance (NMR) spectroscopy

Cell methanol/chloroform/water extracts were made to separate aqueous and organic phases [79]. Methanol/water extracts (containing water soluble compounds) and chloroform extracts (containing insoluble water compounds) and cell culture supernatants were analyzed through NMR spectroscopy. Lyophilization of the aqueous and chloroform extracts was made using SpeedVac Plus system and then organic samples were dissolved in CD_3_Cl and aqueous sample in deuterated water (D_2_O) with 0.04% (v/v) azide and 0.22 mM 3-(trimethylsilyl)propionic-*2,2,3,3-d*4 acid (TSP) as chemical shift reference. For cell culture supernatants, 60μL a solution of 2.2 mM TSP and 0.4% (v/v) azide in D_2_O was added to 540μL of culture media. ^1^H-NMR and ^1^H,^13^C-Heteronuclear single quantum coherence (HSQC)-NMR spectra were obtained at 25°C in an UltrashieldTM 800 Plus spectrometer (Bruker) equipped with a TXI-Z probe and ^13^C-NMR spectra were obtained at 25°C in an UltrashiedTM 500 Plus spectrometer (Bruker) equipped with a ^13^C Dual probe. Spectra were acquired and processed using TopSpin 3.2 software (Bruker) and the assignments were made by resorting to spectral databases: Human Metabolome (HMDB) [80] and Biological Magnetic Resonance Data Bank (BMRB) [81]. Samples were tested in triplicates.

### Cell proliferation assay and calculation of population doubling time (DT)

Cells were collected at 6, 12, 24, 30 and 48h after lactate and/or VEGF plus heparin supplementation. The cell number per mL was calculated using a Bürker counting chamber and cell viability was determined using 0.4% (w/v) trypan blue stain (15250-061, Gibco), at a ratio of 1:4. Six replicates were analyzed for each cell line and culture condition.

Population doubling time (DT) or the time required for a culture to double the number of cells was calculated according to ATCC^®^ Animal cell culture Guide, using the following formula:

$$\mathrm{DT}\;=\;\text{Tln}2/\text{ln}\left(\frac{\text{Xe}}{\text{Xb}}\right)$$

where T is the incubation time in any units, Xb is the cell number at the beginning of the incubation time and Xe is the cell number at the end of the incubation time.

### Calculation of the duration of cell cycle phases

The duration of a particular phase of the cell cycle can be predicted using the following formula [82]:

$$\frac{\text{Tx}}{\text{DT}}\;=\;\frac{\text{ln}\left(\text{FS}+1\right)}{\text{ln}2}$$

where, Tx is the duration of cell cycle phase of interest (e.g. S phase, G2-M phase), DT is the duration of cell cycle (described above) and FS is the fraction of cells in the cell cycle phase of interest (e.g. S phase, G2-M phase), estimated from the DNA content frequent histogram.

The DNA content frequent histogram was carried out using propidium iodide (PI) staining by fluorescence-activated cell sorting (FACS). Cells were collected at DT of each cell line and condition, fixed in 70% ethanol (100983, Merck) and stored at 4°C. Cells were centrifuged at 1500 rpm for 5 min and stained with 100μl PI solution (50μg/mL PI (1001498536, Sigma), 0.1 mg/mL RNase (RN-001, Citogene), 0.05% Triton X-100) and incubated at 37°C for 40 min. After incubation, cells in phosphate-buffered saline (PBS) 1X were centrifuged at 1500 rpm for 10 min at 4°C. The supernatant was discarded and cells were resuspended in 200μl of 0.1% (w/v) bovine serum albumine (BSA) (A9647, Sigma) in PBS 1X and analyzed by FACS (FACScalibur – Becton Dickinson. Data were analyzed using FlowJo (http://www.flowjo.com/) software.

### Cell death analysis by flow cytometry

Supernatants and cells collected were centrifuged at 1200 rpm for 2 min, and resulting pellets incubated with 1μl Alexa Fluor^®^ 488 annexin V (640906, BioLegend) and 1μl propidium iodide (PI) (50μg/mL) in 100μl annexin V binding buffer 1X (10 mM Hepes (pH 7.4) (391333, Millipore), 0.14 M sodium chloride (NaCl; 106404, Merck), 2.5 mM calcium chloride (CaCl_2_; 449709, Sigma) and incubated at room temperature and in dark for 15 min. After incubation, samples were rinsed with 0.1% (w/v) BSA in PBS 1X and centrifuged at 1200 rpm for 5 min. Cells were resuspended in 200μl of annexin V binding buffer 1X. Acquisition was performed in a FACScalibur (Becton Dickinson) and data were analyzed using FlowJo (http://www.flowjo.com/) software.

### Immunofluorescence to evaluate MCT1 and MCT4 in AML cell lines

Cells were collected and centrifuged at 1200 rpm for 2 min, and resuspended in PBS 1X. Cell suspensions (100μl) were transferred to a glass slide by centrifugation in a Shandon CytoSpin III Cytocentrifuge at 1200 rpm for 5 min and then fixed in methanol (CL00.1307.2500, Chem-Lab) for 30 min. Cells were delimitated with a hydrophobic pen (S2002, Dako) and blocking was performed with 0.1% (w/v) BSA in PBS 1X for 30 min at room temperature. Primary antibodies were incubated overnight diluted in 0.1% (w/v) BSA in PBS 1X, 1:50 anti- MCT1 (AB3538P, Millipore); 1:100 anti- MCT4 (SC-50329, Santa Cruz) in a humid chamber. After incubation, slides were rinsed with PBS 1X and then incubated with secondary antibody anti-rabbit conjugated with Alexa Fluor© 488 (A-11034, Invitrogen - Life Technologies Inc) (diluted in 0.1% (w/v) BSA in PBS 1X, 1:1000) for 2 hours, at room temperature. The slides were mounted in VECTASHIELD media with DAPI (4'-6-diamidino-2-phenylindole) (Vector Labs) and examined by standard fluorescence microscopy using a Nikon Instruments Eclipse Ti-S Inverted Microscope (Hamamatsu digital camera C10600 ORCA-R2). Images acquired and processed with NIS-Elements AR-3.2. software and quantified with ImageJ software.

### Western blotting

Total protein extracts were obtained after cell lysis in 20 mM MOPS (pH 6.5), 1% Triton X-100 (v/w) previously supplemented with 1mM Na3VO4, 1mM NaF and 1X protease inhibitors (11836170001, Roche). Protein concentration was determined using standard Bradford protein assay. Then, 100μg protein were separated by electrophoresis in a 12% sodium dodecyl sulfate polyacrylamide gel (SDS-PAGE) and transfered to nitrocelulose membranes (Bio-Rad). Membranes were incubated with anti-MCT1 (1:500; ab179832, Abcam); anti-MCT4 (1:100; SC-1650329, Santa Cruz) and anti-β-actin (1:5000; A5441, Sigma) overnight at 4°C, followed by incubation with horseradish peroxidase (HRP)-conjugated secondary antibodies (anti-mouse; 31430, Thermo Scientific and anti-rabbit; 31460, Thermo Scientific), for 2 hours at room temperature. Membranes were developed with ECL method (Pierce™ ECL Western Blotting Substrate, 32106). Digital images were obtained in ChemiDoc XRS System (Bio-Rad) with Image Lab Software (imagej.nih.gov/ij/) and protein intensity bands were quantified by digital analysis using ImageJ Software (http://rsbweb.nih.gov/ij/). Protein band intensity was normalized to β-actin. Samples were tested in triplicates.

### Agarose gel electrophoresis to analyze LDHs isoenzymes in extracts of AML cell lines

Cells were lysed in 0.01% (w/v) Triton X100 in PBS 1X and centrifuged at 14000 rpm for 10 min at 4°C. Supernatants were collected and stored at -20°C. Protein concentration was determined based on Bradford method, using Bio-Rad protein assay reagent (500-0006, Bio-Rad) through spectotrophometric quantification (595 nm). Equal amounts of total protein (20μg) were analyzed for LDHs isoforms using a procedure for electrophoresis of LDH isoenzyme, using LDH Isoenzymes Electrophoresis Kit (SRE612K, Interlab) according to manufacturer’s protocol in an EasyFix Interlab G26 equipment.

### Immunohistochemistry to detect MCT1 and MCT4 in BM samples from AML patients

Bone marrow (BM) paraffin-embedded tissue blocks from AML patients and patients with normal BM, were obtained retrospectively from the Pathology Department of the Instituto Português de Oncologia de Lisboa Francisco Gentil, EPE (IPOLFG, EPE). Patients’ clinical information was assigned by Hemato-Oncology Laboratory of IPOLFG, EPE. AML were classified according to the 2014 World Health Organization classification that follows FAB classification in which AML are classified according to cells differentiation: Mo- myeloblastic without maturation; M1- myeloblastic with minimal maturation; M2- myeloblastic with maturation; M3- promyelocytic; M4- myelomonocytic; M5- monocytic; M6- erythroleukemia, M7- megakaryoblastic. Clinical evaluation of both MCT1 and MCT4 expression and localization within BM were evaluated by a pathologist.

BM are transported and fixed in acetic acid–zinc–formalin fixative, decalcified in 10% formic acid–5% formaldehyde and processed to paraffin-wax embedding. Paraffin sections (2μm) were at first deparaffinized and re-hydrated and then rinsed with 1X Tris Buffered Saline (TBS) and incubated with primary antibody (anti-MCT1, AB3538P, Millipore and anti- MCT4, SC- 1650329, Santa Cruz) diluted 1:400 and 1:150 respectively, in Bond Primary Antibody Diluent (AR9352, Leica bond) for 1 hour at RT. Subsequently incubation, sections were rinsed 1X TBS and incubated with Peroxidase Block (K4011, Dako) for 10 minutes followed by an incubation with Labelled Polymer-HRP anti-rabbit (K4011, Dako) for 30 minutes at RT, DAB+substrate buffer (K4011, Dako) plus DAB+ chromogen (K4011, Dako) for 8 min and immersed in Mayer’s Hematoxylin for 1 minute. Slides were dehydrated and mounted with Entellan (1079610500, Merck) mounting medium and examined in bright field microscopy (Zeiss). Tonsil was used as negative control, which is completely negative for MCT4 but is positive for MCT1 only in cells that are highly proliferative in the lymphoid germinal centers. Normal uterine cervix was used as positive control, it is positive for MCT1 in squamous cells of the ectocervix and positive for MCT4 in the glandular epithelium of endocervix.

### Relative real-time PCR to quantify MCT1 and MCT4 in BM samples from AML patients

RNA was retrospectively obtained from BM samples, M0-M5 (n=58); M6/M7 (n=13), collected at time of diagnosis of AML patients with available material (Hemato-oncology laboratory (LHO), IPO Lisboa).

cDNA was synthesized from 1μg RNA and reversely transcribed by SuperScript II Reverse Transcriptase (18080-44, Invitrogen), according to the manufacturer’s protocol, in a T3000 thermocycler (Biometra).

Quantitative Real-Time PCR (qRT-PCR) was performed using Power SYBR Green PCR Master Mix (4367659, AB), according to manufacturer’s protocol. Primers for MCT1 (For: 5’GCTGGGCAGTGGTAATTGGA3’; Rev: 5’CAGTAATTGATTTGGGAAATGCAT3’), MCT4 (For: 5’CACAAGTTCTCCAGTGCCATTG3’; Rev: 5’CGCATCCAGGAGTTTGCCTC3’) and housekeeping gene – 18S (For: 5’GCCCTATCAACTTTCGATGGT3’; Rev: 5’CCGGAATCGAACCCTGATT3’) were used. Real-time PCR was carried out in an ABI Prism^®^ 7900HT Sequence Detection System (Applied Biosystems).

For this experiment, cDNA obtained from an RNA pool of six BM samples from hematological disease-free individuals was used as a control.

### Data statistical analysis

Data were analyzed using one-way and two-way ANOVA tests in GraphPad Prism 5 software, and multivariate analysis (Post hoc test Tukey) in SPSS software. Statistically significant changes were determined at the p value <0.05
